# Dysautonomia Following Tetanus, Diphtheria, and Pertussis Vaccine (Tdap): The First Case of Extreme Cachexia Caused by Autoimmune/Inflammatory Syndrome Induced by Adjuvants (ASIA Syndrome) in a Human

**DOI:** 10.3390/medicina57121333

**Published:** 2021-12-06

**Authors:** Or Hen, Paula David, Yehuda Shoenfeld

**Affiliations:** 1 Zabludowicz Center for Autoimmune Diseases, Sheba Medical Center, Tel Hashomer, Affiliated to Tel-Aviv University, Ramat Gan 52621, Israel; or.hen@sheba.health.gov.il (O.H.); paula.david@sheba.health.gov.il (P.D.); 2 Department of Internal Medicine B, Sheba Medical Center, Tel Hashomer, Affiliated to Tel-Aviv University, Ramat Gan 52621, Israel; 3 President of Ariel University, Ariel 40700, Israel

**Keywords:** vaccine, TDaP, autoimmune/inflammatory syndrome induced by adjuvants, autoimmunity, molecular mimicry, aluminum, cachexia, autoantibodies

## Abstract

Autoimmune/inflammatory Syndrome Induced by Adjuvants (ASIA; Shoenfeld’s syndrome) comprehends a group of autoimmune conditions that flourish in genetically predisposed individuals, following an external stimulus by the so-called adjuvants. Many adjuvants were described, such as vaccines, aluminum and other metals, silicone, tattoos, among others. Those conditions entail defined diseases, such as sarcoidosis and Sjogren’s syndrome, and generalized complex symptoms, for example, fatigue, sleep disturbance, orthostatic intolerance, and other dysautonomic manifestations. Those complaints were previously associated with autoantibodies against nervous system autonomic receptors, especially antibeta 1 adrenergic receptor antibodies, suggesting the autoimmune component of the condition. Here we report on a case of an 18-year-old woman who presented with extreme cachexia due to severe dysautonomia caused by the ASIA syndrome induced by the tetanus, diphtheria, and pertussis vaccine (Tdap).

## 1. Case Report

Section 1—An 18-year-old woman presented with severe cachexia, weighing 27.2 kg and a Body mass index (BMI) of 12, with no known history of anorexia nervosa. She was healthy and a professional gymnast until the age of 14, when she got her tetanus, diphtheria, and acellular pertussis vaccine (Tdap). Four days after receiving the immunization, she developed local reactions with tenderness, redness, and swelling at the vaccine injection site as well as daily systemic fevers and headaches. The headaches became worsen with exercises. After one week, the symptoms disappeared, except for a persistent headache. She then presented with episodes of dizziness of 30 s to 5 min in duration, which disturbed her training lessons, and after an episode of syncope with a head trauma, she was forced to leave the activity. The symptoms remained the same until one year later when they became worse. On top of the headaches and dizziness, she experienced arthralgia, especially on her hips, shoulders, and knees; myalgia, extreme fatigue, orthostatic intolerance, mouth ulcers and new food and pollen allergies, symptoms that she still deals with. The patient then consulted a rheumatologist who prescribed her daily hydroxychloroquine under the diagnosis of systemic lupus erythematosus (SLE).

**Figure 1:**
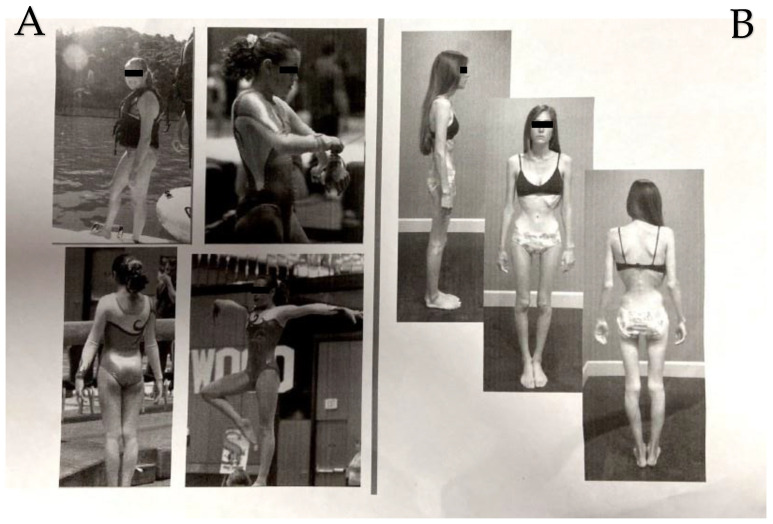
Picture of our patient before (**A**) and after (**B**) receiving the vaccine.

**Figure 2:**
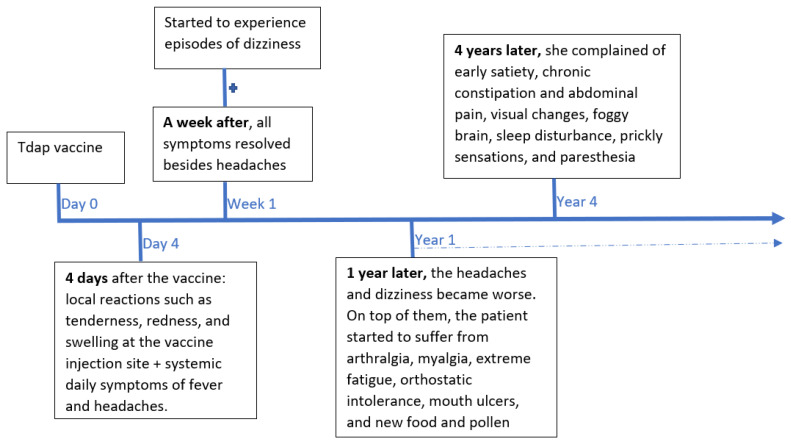
Patient symptoms timeline.

Section 2—Four years after the vaccine, when she presented to us, she also complained of early satiety, chronic constipation and abdominal pain, visual changes, foggy brain, sleep disturbance, prickly sensations, and paresthesia. She is now extremely cachectic (Figure 1), with a weight loss of around 25 kg since the vaccine four years ago. On her examination, she had bradycardia, but apart from that, a normal physical examination, including the neurological examination. Most of her routine, metabolic, hormonal, and immune laboratory workups were between the normal ranges, including ANA, p-ANCA, c-ANCA, antiRo, antiLa (that were not detected), and complement levels. The exceptions were the presence of antihistones, a high immunoglobulin E level, a slightly elevated ESR, and high titers of tetanus antibodies. She was not tested for antineuronal antibodies. Glucose and hemoglobin A1C were normal. Celiac serology and cancer markers were negative. All imaging testing, that included a total body CT scan with and without contrast and a brain magnetic resonance imaging (MRI), were interpreted as normal. Both an optical coherence tomography (OCT) and a Humphrey visual field examination showed no abnormalities. Endoscopic exams did not show absorption disorders or other findings that could explain the clinical picture. A gastric emptying study showed 22% of the normal emptying rate. The psychiatric evaluation did not disclose any mental origin for the eating disorder. She was put on parenteral nutrition with a slow improvement of her weight. She received the diagnosis of autoimmune/anti-inflammatory syndrome induced by adjuvants (ASIA; Shoenfeld’s syndrome) following the Tdap vaccine, with an important component of dysautonomia that explains most of her complaints, including gastroparesis and, consequently, extreme cachexia (Figure 1 and Figure 2).

## 2. Introduction

Autoimmune/inflammatory syndrome induced by adjuvants (ASIA; Shoenfeld’s syndrome) includes a cluster of immune-mediated conditions triggered by the exposure to agents with adjuvant characteristics, usually in genetically predisposed individuals [1]. Some of the conditions are as follows: The postvaccination phenomena and the macrophagic myofasciitis syndrome (MMF) both most likely triggered by aluminum hydroxide as the inciting agent, the Gulf War syndrome (GWS), siliconosis, and the sick building syndrome (SBS) [2,3]. All those syndromes may present autoantibodies and share similar clinical manifestations such as dry mouth, myalgia, myositis, arthralgia, neurological symptoms, cognitive changes, fever, and chronic fatigue syndrome (CFS) [1,4]. In addition, the complaints tend to improve once the triggering agent is removed [1]. For instance, the extraction of silicone breast implants as well as the removal of metal dental and orthopedic implants were shown to improve both the complaints and the autoantibodies/laboratory findings in patients who developed the ASIA syndrome when exposed to them [1,5,6,7,8].

The mentioned syndromes result from an interplay between genetic predisposition and exposure to environmental factors leading to autoimmunity [9,10,11]. A notable example is that individuals with the ASIA syndrome following silicone implants were more likely to have certain genetic haplotypes of the human leukocyte antigen (HLA), including HLA-DR5 and HLA-DQ2 when compared to women with breast implants who were symptom-free [12].

Another well-described trigger for the ASIA syndrome is aluminum, mainly as an adjuvant component in vaccines. It acts as an enhancer of the immune response to immunization, which may provide the structure of its autoimmune reaction pathways [13]. After aluminum salts injection danger-associated molecular patterns, such as uric acid, are released in high concentrations, they form monosodium urate crystals that are phagocytosed by resident cells and disrupt lysosome functions. As a result, cathepsin B is released and can directly or indirectly activate the intracellular Nalp3 inflammasome system and caspase-1. In this way, aluminum stimulates the production and secretion of various cytokines such as IL-1b; IL-18, and IL-33 [13]. In addition to the local immune stimulation, adjuvants establish physical protection to antigens and help in antigen translocation to the regional lymph nodes. Thus, a longer exposure of the immune system to the antigen provides enhanced production and activation of both the B and T cells, mounting a more robust immune response [13,14,15]. Notably, the adjuvant aluminum may be contained in immune complexes produced following vaccination [14,16].

Interestingly, other peculiar triggers to ASIA have been proposed, such as tattoo ink [17], dental and orthopedic metal implants [6,18], and polypropylene mesh implantation [19].

The Tetanus Toxoid, Reduced Diphtheria Toxoid, and Acellular Pertussis Vaccine (Tdap)—Adacel^®^—was first approved by the Food and Drugs Administration (FDA) in 2005 for adolescents and adults of 11–64 years of age [20]. The Adacel^®^ vaccine contains noninfectious tetanus, diphtheria, and pertussis proteins; aluminum phosphate, 2-phenoxyethanol, and residual amounts of formaldehyde and glutaraldehyde. It does not contain preservatives [21]. The most commonly described adverse reactions to Tdap are injection site pain (77.8%); headache (43%); myalgia (30.4%); fatigue (30.4%); injection site swelling (20.9%); injection site erythema (20.8%); chills (15.1%); arthralgia (11%); nausea (13.3%); lymph node enlargement (6.6%); diarrhea (10.3%); fever (5%); vomiting (4.6%) and rash (2.7%) [20].

## 3. Discussion

Our patient developed some of the most frequent symptoms of the ASIA syndrome, including arthralgia, chronic fatigue syndrome, paresthesia, dizziness, sleep disturbances, headaches, and difficulty focussing for a long time as well as orthostatic intolerance, starting some days after receiving the Tdap immunization and that still remained after a period of almost four years [22,23]. Vaccines, especially those containing aluminium are one of the most commonly described adjuvants for ASIA. In one cohort study with 300 ASIA patients, 119 of them had vaccines as the only exposure factor identified previous to the development of the symptoms, including the Tdap immunization [23]. In the updated study, with a cohort of 500 ASIA syndrome patients, almost half of the subjects had a clinical manifestation after vaccine exposure, and 8.4% of them were exposed to the Tdap vaccine [24].

There are several possible pathways through which vaccines may induce immune reactions. It may include infection-like mechanisms, such as possible molecular mimicry between the vaccine’s components and human peptides resulting in a cross-reaction; epitope spreading, in which vaccine antigens locally activate antigen-presenting cells, accelerating an underlying autoimmune process; and the polyclonal activation of B cells, increasing the release of proinflammatory molecules, such as cytokines, and, consequently, permitting the activation of autoreactive T cells [25]. Moreover, the presence of adjuvants in the vaccines, such as aluminum, that are able to enhance the host immune response, have an important role in ASIA syndrome development following immunization [26].

The Tdap, as previously mentioned, contains aluminum. The fact that our patient presented with a remarkably high tetanus antibodies titer supports the theory of an exaggerated immune response to the vaccine. Interestingly, in a murine model, antibodies against tetanus toxoid cross-reacted with beta-glycoprotein I, suggesting a potential molecular mimicry between those peptides [27]. Regarding the other laboratory findings in our patient, most of the results were within the normal range, except for a positive anti-histone and slightly elevated ESR. We have not found in the literature cases of antihistone positivity following vaccination or other adjuvants of the ASIA syndrome. However, it reinforces the possibility of the autoimmune etiology of our patient’s symptoms.

Our patient developed the typical ASIA symptoms at a later point of the disease course. Other cases with late-onset classical ASIA syndrome were previously reported [28,29].

In addition to the common ASIA symptoms, the patient we described here had a significant weight loss, with a decrease of more than 50% of her original body weight (Figure 1) after receiving the Tdap. She complained about delayed gastric emptying sensation, which was confirmed by a nuclear gastric study. The Vaccine Adverse Event Reporting System (VAERS) is the FDA’s and Centers for Disease Control (CDC)’s program for controlling vaccine safety and adverse effects that collects information worldwide. When looking at VAERS data for complaints of gastric hypomotility following vaccination, 11 cases were reported, one of them being after the Tdap [30]. In the same database, we found nine cases of patients with weight loss after receiving the Tdap [30]. Interestingly, in a published case of the ovine ASIA syndrome following vaccination against bluetongue, extreme cachexia was described as a chronic phase of the ASIA syndrome that was very similar to what we saw in our patient [31].

Altogether, most of our patient’s manifestations could be explained by dysautonomia, including headaches, orthostatic intolerance, gastroparesis and consequent weight loss, paresthesia, and sleep disturbances. Similar symptoms were described after the HPV vaccination and hepatitis B immunization [32,33]. In a cohort of 93 patients after the HBV vaccine with these clinical manifestations, 80% of those patients were also found to have high levels of different autoantibodies [32]. Autoimmune neurosensory dysautonomia has been proposed as an explanation for those symptoms, commonly also resulting in small fiber neuropathy and the presence of autoantibodies against G-coupled protein autonomic receptors, such as muscarinic, nicotinic, and adrenergic [34]. Indeed, patients with orthostatic intolerance and postorthostatic tachycardia syndrome were also found to be positive to those autoantibodies [35,36,37,38].

Individuals with the ASIA syndrome triggered by silicone breast implants were shown to present with a disbalance in the autoantibodies against G-coupled protein receptors when compared to healthy controls [34]. Those patients have a variety of complaints that include dysautonomic symptoms such as chronic fatigue, sleep disturbances, memory loss, cognitive impairment, generalized pain, depression, anxiety, among others [7,39]. Recently, it was demonstrated that the dysautonomic symptoms are particularly related to a decrease in the titers of antibeta 1 adrenergic receptor antibodies levels when compared to the controls [39].

Those autoantibodies could be a marker to identify autoimmune dysautonomia due to the ASIA syndrome after silicone implants and potentially also following other triggers, such as in our patient [39].

## 4. Conclusions

Here we presented an interesting case of an 18-year-old woman who developed the ASIA syndrome after receiving the Tdap vaccine. Her symptoms were mainly generic and autonomic, which slowly evolved in severity, and, after four years, reached the point of a loss of more than 50% of her original body weight due to gastroparesis. Notably, a similar case of a chronic phase of the ASIA syndrome with severe cachexia was previously described following vaccination in sheep [31]. Dysautonomia has been associated with autoimmunity and the ASIA syndrome. [7,32,33,34]. In addition, autoimmune dysautonomia was linked to autoantibodies against G-coupled protein receptors, in particular with antibeta 1 adrenergic receptor antibody, in patients with silicone breast implants [39]. This antibody could represent a potential tool for recognizing cases of ASIA syndrome following different adjuvant stimuli. To our knowledge, this is the first case described in the literature of the ASIA syndrome presenting as extreme cachexia due to severe dysautonomia.

## Data Availability

Not applicable.
